# Supplementary material for: Poor Long-Term Renal Allograft Survival in Patients with Chronic Antibody-Mediated Rejection, Irrespective of Treatment—A Single Center Retrospective Study
Source: J Clin Med. 2021 Dec 30;11(1):199. doi: 10.3390/jcm11010199 (PMC8745558; doi:10.3390/jcm11010199)
Supplement: Supplementary file 1 [file jcm-11-00199-s001.zip › jcm-1520546-supplementary.pdf]

Supplementary

Table S1. univariate Kaplan-Meier analysis of severity of Banff lesion score on 5-year death-censored graft survival after diagnosis of cAMR.

Score of Banff lesions	death-censored graft survival	Log rank，p-value
g0 (*n* = 44)	31.80%	
g1(*n* = 11)	36.40%	0.74
cg1 (*n* = 14)	35.70%	
cg2-3 (*n* = 41)	31.70%	0.92
mm0-1 (*n* = 48)	35.40%	
mm2-3 (*n* =7)	14.30%	0.22
v0-1 (*n* = 51)	33.30%	
v2-3 (*n* = 4)	25.00%	0.15
ah0-1 (*n* = 8)	37.50%	
ah2-3 (*n* = 47)	31.90%	0.93
cv0-1 (*n* = 23)	43.50%	
cv2-3 (*n* = 32)	25.00%	0.17
ai0-1 (*n* = 41)	34.10%	
ai2-3 (*n* = 14)	28.60%	0.41
at0-1 (*n* = 51)	33.30%	
at2-3 (*n* = 4)	25.00%	0.36
ci0-1 (*n* = 46)	34.80%	
ci2-3 (*n* = 9)	22.20%	0.68
ct0-1 (*n* = 45)	33.30%	
ct2-3 (*n* = 10)	30.00%	0.89
ptc0-1 (*n* =51)	35.30%	
ptc 2-3 (*n* = 4)	0.00%	0.05
